# Evaluating isoprenol production using the IPP-bypass pathway in the oleaginous yeast *Rhodosporidium toruloides*

**DOI:** 10.1186/s13068-026-02750-w

**Published:** 2026-03-03

**Authors:** Valentina E. Garcia, Gina M. Geiselman, Hee Jin Hwang, Yan Chen, Jennifer W. Gin, Jack McFadden-Mitchell, Veronica Montgomery, Ramu Kakumanu, Shubhasish Goswami, Joshua McCauley, Paul A. Adamczyk, Edward E. K. Baidoo, Christopher J. Petzold, Blake A. Simmons, Taek Soon Lee, John M. Gladden

**Affiliations:** 1 Joint BioEnergy Institute, Emeryville, CA 94608 USA; 2 Department of Biomaterials and Biomanufacturing, Sandia National Laboratories, Livermore, CA 94550 USA; 3 Biological Systems and Engineering Division, Lawrence Berkeley National Laboratory, Berkeley, CA 94720 USA; 4 San Diego State University, San Diego, CA 92182 USA

**Keywords:** Isoprenol, Rhodosporidium toruloides, IPP-bypass pathway, Phosphatase, Advanced aviation fuel

## Abstract

**Background:**

To strengthen the national energy supply, there is an increasing demand for domestically generated aviation fuels. Bio-derived advanced aviation fuels offer the opportunity to meet this domestic need while presenting a unique opportunity to investigate the production of novel aviation fuels. Isoprenol, a chemical precursor to such novel fuels, has been shown to be a biologically producible compound in model organisms, but its bio-producibility needs to be further explored in organisms more compatible with industrial bioproduction.

**Results:**

In this work, we evaluate isoprenol production using the promising bioproduction yeast, *Rhodosporidium toruloides*. First, we show successful isoprenol production using the IPP-bypass pathways most successful in laboratory strains of *E. coli* and *S. cerevisiae*. Next, we demonstrate that increased flux through the mevalonate pathway only modestly increases isoprenol titers. Using proteomics, we identified a potential bottleneck in production at the final step in the IPP-bypass pathway and explored alternative enzymes for this step. Finally, the top three strains of *R. toruloides* were evaluated in sorghum hydrolysates generated using cholinium lysinate. Through this work, 93.1 mg/L of isoprenol was produced in mock medium and 27.3 mg/L in sorghum hydrolysates.

**Conclusion:**

Together these results lay the foundation for future work for the production of isoprenol from bioproduction crops.

**Supplementary Information:**

The online version contains supplementary material available at 10.1186/s13068-026-02750-w.

## Background

The urgent need for a secure national energy supply is driving efforts to develop a robust biomanufacturing economy. In developing these advanced manufacturing routes, there is the opportunity to explore novel compounds with improved properties over what is currently available. This opportunity is especially present for aviation fuels where novel formulations could improve performance, be easier to produce at scale, and increase energy efficiency [1–3].

As high energy molecules are difficult to generate biologically, focus has been placed on the bioproduction of versatile chemical precursors that can be chemically converted into aviation fuels. For example, 1,4-Dimethylcyclooctane (DMCO) has exceptional fuel properties that make it a prospective high-performance jet fuel blend stock replacement for Jet A, including higher energy density and higher volumetric net head of combustion [4]. DMCO can be catalytically produced from isoprene, a component of natural rubber, that is unfortunately challenging to produce through fermentation due to its extreme volatility. However, the precursor to isoprene, isoprenol (3-methylbut-3-en-1-ol), is more amenable to bioproduction and could therefore act as a biologically produced precursor to DMCO. The current cost of isoprenol through bioproduction is high, $5.14/L-gasoline equivalent, and significant optimization is needed across the production chain to lower this cost to theoretically achievable levels, $0.62/L-gasoline equivalent [5].

**Fig. 1:**
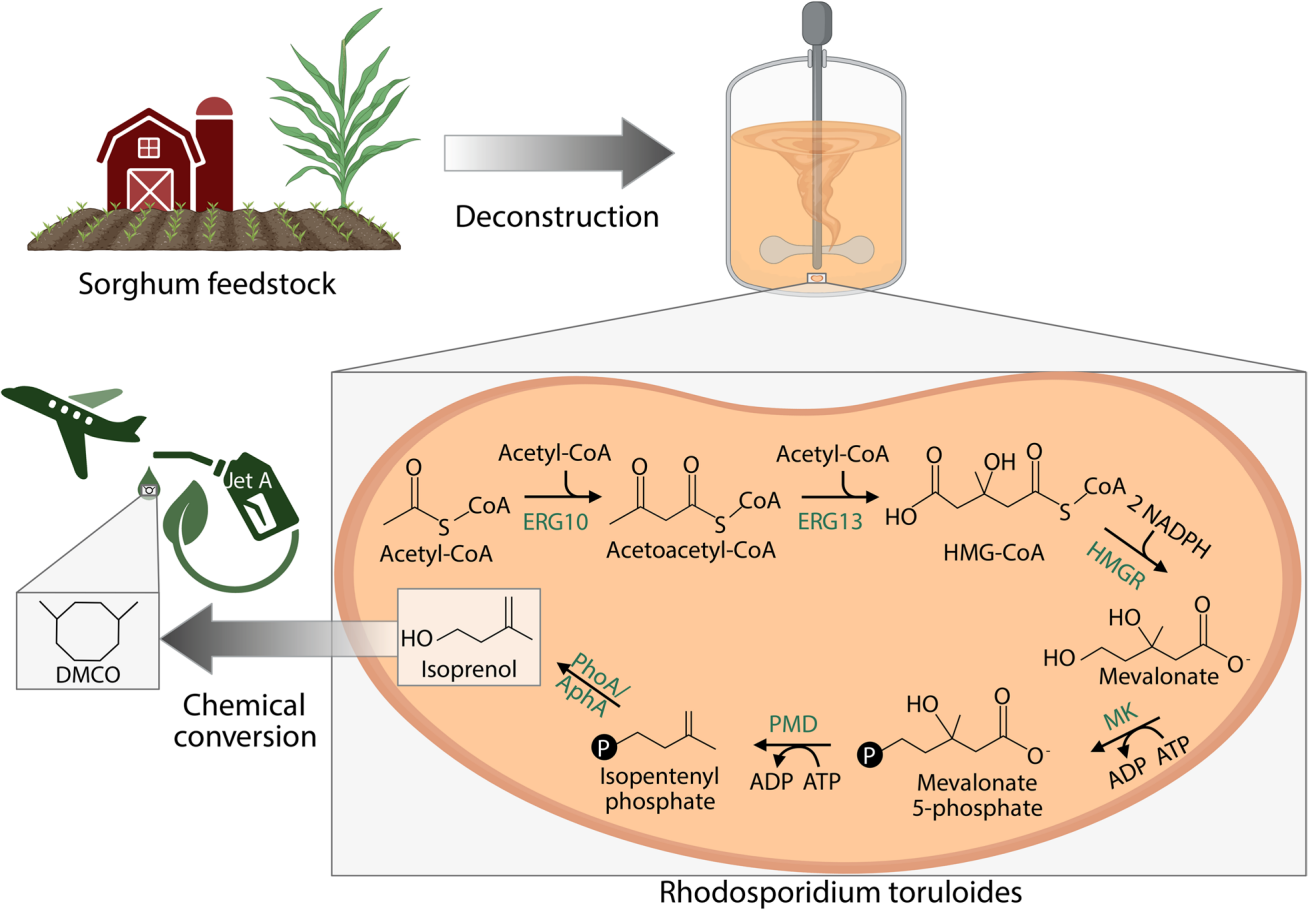
Overview of isoprenol production from a plant-based feedstock for DMCO conversion using the IPP-bypass pathway in R. toruloides. Figure created using BioRender

The bioproduction of isoprenol has been demonstrated through two distinct metabolic pathways, an amino acid derived pathway [6] and the mevalonate pathway. The foundational mevalonate pathway deployed in *E. coli* employs 7 heterologous enzymes starting at the conversion of acetyl-CoA to mevalonate (MVA), proceeds through the production of isopentenyl diphosphate (IPP), which is dephosphorylated into isopentenyl phosphate (IP) and, again into isoprenol. The mevalonate pathway was further developed into the IPP-bypass (IPPb) pathway that circumvents the accumulation of toxic IPP by directly converting MVA monophosphate to IP using a promiscuous mutant of a mevalonate pyrophosphate decarboxylase, Erg19 (annotated PMD) [7] (Fig. 1).

Implementation of the IPPb pathway with additional engineering efforts has further improved the titers, rates, and yields of isoprenol production across host systems. In *E. coli*, systematic CRISPR interference tuning of the IPPb pathway has achieved isoprenol titers of up to 12.4 g/L in fed-batch cultivation [8]. Similar optimization in *P. putida* has yielded titers of 3.5 g/L under fed-batch conditions [9]. For *C. glutamicum*, a combination of pathway engineering and strain background optimization achieved titers up to 1.25 g/L in flask culture [10]. Finally, in *S. cerevisiae*, efforts to optimize the IPPb pathway have resulted in isoprenol titers of 383.1 mg/L in flask cultures [11]. Unfortunately, many of these hosts struggle with production in a biomanufacturing setting due to the complex nature of plant derived hydrolysates[12–16] or need to be engineered to utilize the diversity of carbon sources present [17, 18].Other hosts have proven more successful at producing high titers of terpenes in more relevant settings[19]. Biomanufacturing compatible hosts capable of producing industrially relevant titers need to be identified for isoprenol production.

*Rhodosporidium toruloides*, also known as *Rhodotorula toruloides* [20], is a promising microbial host for bioproduction due to several advantageous characteristics. Notably, *R. toruloides* can robustly metabolize various lignocellulosic hydrolysates, enabling the use of abundant and cost-effective biomass feedstocks. It also demonstrates resilience under industrially relevant fermentation conditions, including tolerance to low pH and the complexity of lignocellulosic hydrolysates. Relating to isoprenol production, *R. toruloides* naturally accumulates high levels of lipids and terpenoids, indicating strong metabolic flux through the mevalonate pathway [21–31]. Additionally, *R. toruloides* is already a demonstrated host for the production of non-native terpenes and has reached production titers up to 20.8 g/L of bisabolene [32]. Collectively, these characteristics make *R. toruloides* a promising host organism for engineering a robust, scalable, and economically viable isoprenol production process.

Here, we deploy the IPPb pathway in *R. toruloides* for the production of isoprenol (Fig. 1). In doing so, we demonstrate production of isoprenol up to 93.1 mg/L in a sugar-rich medium and 27.3 mg/L using sorghum-derived hydrolysates. Through this work we identify key areas for further optimization using proteomic analysis and evaluating isoprenol degradation by *R. toruloides*.

## Methods

### Strain generation and plasmid information

Strains throughout this study were generated using  *Agrobacterium*-mediated transformation (ATMT) performed as described in the following text. Twenty µL of electrocompetent *Agrobacterium tumefaciens* EHA 105 cells was combined with 1 µg of plasmid DNA in a 0.2 cm electroporation cuvette (BioRad, Hercules, CA, USA; 1,652,086). The mixture was electroporated using a Gene Pulser Xcell electroporation module (BioRad) set at 2.5 kV, 25 µF, 400 Ω. Immediately following electroporation, cells were recovered in 1 mL of LB broth (Spectrum Chemicals, New Brunswick, NJ, USA; 743-29238-10) and incubated for 1 h at 30 °C. Cells were plated onto LB agar (Thermo Fisher Scientific, Waltham, MA, USA; DF0140010) plates containing 50 µg/mL kanamycin and incubated at 30 °C for at least 2 days. Simultaneously, *R. toruloides* strains for transformation were prepared by streaking from glycerol stocks onto YPD (Thermo Fisher Scientific; DF0428-17-5) agar plates and incubated at 30 °C for at least 2 days.

Individual colonies from successfully transformed *A. tumefaciens* were inoculated into 10 mL of LB broth containing 50 µg/mL of kanamycin, while *R. toruloides* colonies were inoculated into 10 mL YPD medium using 25 mL culture tubes (VWR, Radnor, PA, USA; 10545-950). All cultures were incubated overnight at 30 °C with shaking at 200 RPM. The next morning, *A. tumefaciens* cultures were diluted to an OD_600_ of 0.5 in 10 mL of LB broth and grown as above until reaching the OD_600_ of approximately 1. Cells were pelleted by centrifugation at 3220 RCF for 15 min (Eppendorf Centrifuge 5810), the supernatant was discarded, and the cells were resuspended in 10 mL induction medium (1 g/L NH_4_Cl, 300 mg/L MgSO_4_ 7H2O, 150 mg/L KCl (Thermo Fisher Scientific; P267-500), 10 mg/L CaCl_2_ (VWR; 0556), 750 µg/L FeSO_4_ 7H_2_O (Thermo Fisher Scientific; AC423731000), 144 mg/L K_2_HPO_4_ (VWR; 0705), 48 mg/L NaH_2_PO_4_ (Thermo Fisher Scientific; BP329), 2 g/L D-Glucose, 10 mg/L thiamine (Sigma-Aldrich, St. Louis, MO, USA; T4625), 20 mg/L acetosyringone (Sigma-Aldrich; D134406), and 3.9 g/L MES (Sigma-Aldrich; 69892), adjusted to pH 5.5 with KOH). On the same day, *R. toruloides* cultures were diluted in fresh YPD to varying OD_600_ concentrations. Both the activated *A. tumefaciens* and *R. toruloides* cultures were incubated at 30 °C with shaking for 24 h.

After the incubation, cells equivalent to 1 OD_600_ of *R. toruloides* were pelleted and resuspended in 1 mL of activated *A. tumefaciens* culture. This mixture was concentrated onto a 0.45 µm membrane filter (EMD Millipore, Burlington, MA, USA; HAWP04700). The filter was placed on induction media plates with 2% agar and incubated at 26 °C for 2–3 days (wild type and dCK strain) or 5–6 days (MVA002 or MVA003). Following incubation, cells from the filter were resuspended by vortexing in 5 mL YPD medium in a 50 mL conical tube. Cells were pelleted, resuspended in 1 mL of YPD, and plated onto YPD agar plates supplemented with 300 µg/mL cefotaxime (TCI, Portland, OR, USA; C2224) and 300 µg/mL carbenicillin (Sigma-Aldrich, C1389-5G) to eliminate surviving *A. tumefaciens* present, and 100 µg/mL nourseothricin to select for successful *R. toruloides* transformants (Werner Bioagents, Jena, Germany; 5.004.000). For wild-type and dCK strains, 100 µL of the suspension was plated, whereas the entire 1 mL suspension was plated for MVA002 and MVA003 strains. Plates were incubated at 30 °C for 2–4 days until colonies appeared.

IFO0880 (also known as NBRC0880), obtained from the Biological Resource Center, NITE (NBRC), Japan, was used as the wild-type *R. toruloides* strain. All plasmids used for ATMT transformation were derived from pGI2 [33]. Plasmids were either synthesized commercially (Genscript, Piscataway, NJ, USA) or constructed using NEBuilder HiFi DNA Assembly (NEB, Ipswich, MA, USA; E5520S). All coding sequences were codon optimized specifically for *R. toruloides* using GenScript’s proprietary codon optimization method that uses the codon usage table provided in Additional File 1. For all isoprenol plasmids, a nourseothricin resistance marker was included for selection.

Four base strains were transformed throughout this study, wild type, dCK, MVA002, and MVA003. To generate MVA002, plasmid pPA193C, containing an acetyl-CoA acetyltransferase (ERG10 UniProt ID: P41338), a hydroxymethylglutaryl-CoA synthase (ERG13, UniProt ID: P54839), and a 3-hydroxy-3-methylglutaryl-coenzyme A reductase 1 (HMGR, UniProt ID: P12683) all from *S. cerevisiae,* was randomly integrated into the genome of *R. toruloides* IFO0880 via ATMT. A visual increase in red pigment from carotenoids was used to identify resulting strains with increased mevalonate flux. In more detail, after pPA193C transformation, colonies were first recovered under antibiotic selection and then screened by visual comparison of colony pigmentation. To more clearly distinguish differences in colony pigmentation, we performed this screening on agar plates with yeast nitrogen base without amino acids (Sigma-Aldrich, St. Louis, MO, USA; Y0626) and complete supplement medium (MP Biomedicals, Irvine, CA, USA; 114500022). Among the colonies on the same plate, we selected the transformant showing the darkest red coloration relative to the others and designated this strain as MVA001. Mevalonate concentrations were then measured and compared to wild type as detailed below . The hygromycin resistance-thymidine kinase (HYG-TK) marker cassette was subsequently recycled from MVA001 by counterselection using 5-fluoro-2'-deoxyuridine (Sigma-Aldrich, F0503-100MG) as performed previously [32], generating the marker-free strain MVA002.

dCK and MVA003, derived from WT and MVA002, respectively, contain a knockout of a putative choline kinase protein (MycoCosm Rhodosporidium toruloides IFO0880 v4.0 Portal Protein ID: RTO4_15982; sequence provided in Additional File 2) and were generated using a lithium acetate (LiAc) transformation protocol similar to what has been previously described [34]. WT and MVA002 were transformed with a linear fragment containing 1000 bp 5’ of RT04_15982, a *TUB2* promoter driving a HYG-TK cassette followed by 192 bp 3’ of RT04_15982 was integrated into the RTO4_15982 locus. To generate the linear fragment the plasmid dCK_RTO415982_del was digested with PVUII (Thermo Fisher Scientific, Waltham, MA; FD0634). Parent cultures were streaked onto YPD agar plates and individual colonies were grown in 10 mL YPD overnight at 30 °C with shaking at 200 RPM. Cultures were then diluted and regrown to an OD_600_ of 0.8. Cultures were centrifuged at 4000 RCF for 5 min, washed twice with water and once with 150 mM LiAc (Sigma-Aldrich). Cells were resuspended in 240 µL 50% PEG 4000, 54 µL 1 M LiAc, 10 µL salmon sperm DNA (Thermo Fisher; 15632011), and 56 µL linearized DNA (1 mg total). Cells were incubated at 30 °C for 1 h, supplemented with 34 µL DMSO, and incubated at 37 °C for 5 min. Cells were centrifuged and washed once with YPD before being grown overnight in 5 mL YPD. Overnight cultures were plated on YPD with 50 µg/mL Hygromycin B and grown for 2–3 days at 30 °C. Colonies were screened for a deletion by colony PCR amplifying the first 460 base pairs of the annotated coding region for RTO4_15982 (forward: ATGGCTCCAGTGTCTCC reverse: CAGTCGACTGGTAGCAC). Knockout colonies were further verified by PCR amplifying the region surrounding the gene encoding RTO4_15982 starting 1086 base pairs 5’ and ending 1001 base pairs 3’ of the annotate coding region (forward primer: GCTGGTCGTTCAGGAAG reverse primer GTATGGTATGATTCGTCGC).

Sequencing was performed on MVA003 by Plasmidsaurus Inc. (Louisvelle, KY, USA). To validate ERG10 and HMGR genomic integration the raw fastq file was QC filtered using filtlong (version: v0.2.1, Docker image: quay.io/biocontainers/filtlong:0.2.1–hdcf5f25_4), with a minimum length of 1 kb and mean_q_weight set to 10 to prioritize read quality while discarding the worst 5% of reads. The resulting filtered reads were then mapped to the reference sequences for each gene using minimap2 (version: 2.29-r1283, Docker image: quay.io/biocontainers/minimap2:2.29–h577a1d6_0). Mapped reads were sorted, indexed, and sequencing depth calculated using samtools (version: 1.22, Docker image: quay.io/biocontainers/samtools:1.22–h96c455f_0).

The plasmid sequences used in the work are available in the supplemental material and at the Joint BioEnergy Institute Public Inventory of Composable Elements (https://public-registry.jbei.org/ Accession numbers provided in Additional File 1 Table 1).

### Media composition and hydrolysate generation

The sugar-rich isoprenol production medium used in this study had a carbon-to-nitrogen ratio of 40:1. Unless stated otherwise, it contained 138.7 g/L glucose, 73.8 g/L xylose, 100 µM iron (II) sulfate, 0.79 g/L complete supplement medium, 1.7 g/L yeast nitrogen base without ammonium sulfate and amino acids (Sigma-Aldrich, Y1251-100G), 10.12 g/L ammonium sulfate, and 100 mM sodium phosphate buffer (pH 6.5). The medium was freshly prepared one day prior to use by combining sterile-filtered (0.22 µm) stock solutions of each component.

Sorghum hydrolysates were generated as previously described [35, 36]. In brief, 15 wt% biomass with 10 wt% Cholinium lysinate ([Ch][Lys]; Iolitec Inc., Tuscaloosa, AL, USA) and 75 wt% water was heated at 140 °C for 3 h in a 10 L Parr reactor (Parr Instrument Company, Moline, IL, USA; 4555–58). Post pretreatment, 10 M HCl was added to adjust the pH of the biomass slurry to 5. Subsequently, a commercial enzyme mixture from Novozymes (Franklinton, NC, USA) containing Cellic CTec3 and HTec3 (9: 1 v/v), was added to the biomass slurry at a concentration of 10 mg enzyme per g biomass to carry out saccharification at 50 °C for 72 h at 48 RPM in the same Parr vessel. After hydrolysis, liquid samples were collected and centrifuged at 8000 RPM for 20 min and the supernatant was filtered using 0.45 μm Rapid flow centrifuge filters (Thermo Fisher) for HPLC analysis.

### Mevalonate intermediates analysis

Wild type *R. toruloides* and engineered strain MVA001 were first cultured overnight in YPD medium, then transferred into GX5AS medium (60 g/L glucose, 30 g/L xylose, 5 g/L ammonium sulfate, 100 mM potassium phosphate buffer at pH 6, 400 μg/L thiamine hydrochloride, 400 μg/L pyridoxine hydrochloride, 100 μg/L FeSO_4_, and 1 mM Na_2_SO_4_) in a 48-well flower plate (Beckmen Coulter, Brea, CA, USA; M2P-MTP-48-OFF). Samples were collected on days 4 and 7 by centrifugation at 16,000 RCF for 3 min at room temperature, and immediately quenched in liquid nitrogen.

Intracellular metabolites were extracted using a previously established methanol–chloroform extraction method [25]. Briefly, cell pellets were resuspended in 300 µL cold methanol and vortexed thoroughly. Next, 300 µL chloroform was added, followed by vortexing, and 150 µL water was added and vortexed again. Samples were centrifuged at 14,000 RCF for 10 min at 4 °C, after which the upper aqueous layer was collected. Water was added to the collection fraction to achieve a final methanol-to-water ratio to 1:1. The resulting samples were filtered through Amicon 3 K molecular weight cutoff filters (Millipore Sigma; UFC500308) prior to analysis. Metabolites were analyzed via the LC–MS method described previously (Amer et al., 2022).

### Isoprenol production evaluation

Fifteen colonies from each transformation were recovered from glycerol stocks by streaking onto YPD agar plates supplemented with 300 µg/mL cefotaxime, 300 µg/mL carbenicillin, and 100 µg/mL nourseothricin, then incubated at 30 °C for 2 days. To ensure isolated single colonies, each resulting colony was re-streaked onto fresh selective YPD agar plates containing the same antibiotics and grown at 30 °C for an additional 3 days.

Single colonies were inoculated into 500 µL YPD medium in sterile 96-well clear V-bottom 2 mL polypropylene deep-well plates (Corning, Corning, NY, USA; 3960) and incubated at 30 °C with shaking at 999 RPM in an Infors (Switzerland) Multitron shaker for 2 days. Subsequently, 100 µL of each culture was transferred into 10 mL of isoprenol production medium in 25 mL culture tubes (VWR; 10,545–950) and incubated at 22 °C with shaking at 200 RPM in an Infors Multitron shaker.

At 6 and 12 days post-inoculation, 600 µL samples were collected into 1.5 mL Eppendorf tubes for isoprenol quantification, proteomics analysis, and OD_600_ measurement. For OD_600_ determination, 2 µL of each culture sample was diluted into 198 µL of water in clear 96-well plates, and absorbance was measured using a Spectra Max Plus 384 microplate reader (Molecular Devices).

### Isoprenol metabolism assay

The ability of *R. toruloides* to catabolize isoprenol was evaluated using the isoprenol production medium described above with or without glucose and xylose. Medium (10 mL) containing isoprenol concentrations of 0, 0.25, 0.5 or 1 g/L of isoprenol was dispensed into 25 mL culture tubes (VWR; 10545–950). For assays involving cells, a starter culture of wild-type *R. toruloides* was grown in YPD medium to a max OD_600_ of 1, then concentrated to an OD_600_ of 10. Each relevant culture tube was inoculated with 100 µL of this concentrated cell suspension, resulting in a starting OD_600_ of 0.1. Cultures were incubated at 22 °C with shaking at 200 RPM in an Infors Multitron shaker.

Cell growth (OD_600_) was measured at days 2, 4, 6, 10, and 14. For the OD_600_, samples were diluted as needed to a final volume of 200uL and measured in a 96-well plate using the Spectra Max Plus 384 microplate reader (Molecular Devices, San Jose, CA, USA). Samples for isoprenol quantification were collected on day 14. To compare isoprenol loss between conditions containing cells with and without sugar, the concentration with cells was normalized by dividing by the mean concentration without cells and multiplying by 100. Data were plotted using GraphPad Prism (Dotmatics, Boston, MA, USA) software.

### Isoprenol quantification

490 µL of well-mixed cell culture was combined with 490 µL of ethyl acetate containing 30 mg/L of 1-butanol as an internal standard. The extraction mixture was vortexed for 10 min at room temperature using a Vortex-Genie 2 (Scientific Industries, Inc, Bohemia, NY, USA) equipped with a Vortex adaptor-60 (Thermo Fisher; AM10014), set at a shaking intensity between levels 8 and 10. Samples were centrifuged at 20,328 RCF for 5 min using a Centrifuge 5424 (Eppendorf, Germany) to separate the phases. Subsequently, 200 µL of the upper organic layer was transferred to HPLC vials fitted with small-volume inserts (Agilent, Santa Clara, CA, USA; 5183-2088). Extracted samples were stored at −80 °C until analysis. For isoprenol quantification, samples were analyzed using an Agilent GC 8890 gas chromatograph equipped with a flame ionization detector (FID), a 15 m × 0.25 mm × 0.25 µm DB-WAX UI column (Agilent; 122–7012), and an automated liquid handler (Agilent; 7693A). Each sample (1 μL) was injected in splitless mode. Helium was used as the carrier gas at a flow rate of 2.2 mL/min, with injector and detector temperatures set at 250 °C and 300 °C, respectively. Isoprenol concentrations were determined by comparison to a standard curve generated each run using 97% 3-Methyl-3-buten-1-ol (Sigma-Aldrich; 129402-100 mL) prepared in ethyl acetate containing 30 mg/L of 1-butanol as an internal standard. Data analysis was performed using Open Lab software (Agilent).

### Proteomics analysis

On day 6 fermentation, 107.5 µL of culture was spun down in PCR-tubes or 96-well plates and the supernatant was subsequently removed. Cell pellets were then frozen at −80 °C until further processing. Protein was extracted from cell pellets and tryptic peptides were prepared by following established proteomic sample preparation protocol [37]. Briefly, cell pellets were resuspended in Qiagen P2 Lysis Buffer (Qiagen, Germany; 19052) to promote cell lysis. Proteins were precipitated with addition of 1 mM NaCl and 4 × vol acetone, followed by two additional washes with 80% acetone in water. The recovered protein pellet was homogenized by pipetting mixing with 100 mM ammonium bicarbonate in 20% methanol. Protein concentration was determined by the DC protein assay (BioRad). Protein reduction was accomplished using 5 mM tris 2-(carboxyethyl)phosphine (TCEP) for 30 min at room temperature, and alkylation was performed with 10 mM iodoacetamide (IAM; final concentration) for 30 min at room temperature in the dark. Overnight digestion with trypsin was accomplished with a 1:50 trypsin:total protein ratio. The resulting peptide samples were analyzed on an Agilent 1290 UHPLC system coupled to a Thermo Scientific Orbitrap Exploris 480 mass spectrometer for discovery proteomics [38]. Briefly, peptide samples were loaded onto an Ascentis® ES-C18 Column (Sigma-Aldrich; 53306-U) and were eluted from the column by using a 10 min gradient from 98% solvent A (0.1% FA in H_2_O) and 2% solvent B (0.1% FA in ACN) to 65% solvent A and 35% solvent B. Eluting peptides were introduced to the mass spectrometer operating in positive-ion mode and were measured in data-independent acquisition (DIA) mode with a duty cycle of 3 survey scans from m/z 380 to m/z 985 and 45 Tandem mass spectrometry (MS^2^) scans with precursor isolation width of 13.5 m/z to cover the mass range. DIA raw data files were analyzed by an integrated software suite DIA-NN [39]. The database used in the DIA-NN search (library-free mode) was *R. toruloides* latest UniProt proteome FASTA sequences plus the protein sequences of the heterologous proteins and common proteomic contaminants. DIA-NN determines mass tolerances automatically based on first pass analysis of the samples with automated determination of optimal mass accuracies. The retention time extraction window was determined individually for all MS runs analyzed via the automated optimization procedure implemented in DIA-NN. Protein inference was enabled, and the quantification strategy was set to Robust LC = High Accuracy. Output main DIA-NN reports were filtered with a global false discovery rate set at 0.01 (FDR <  = 0.01) on both the precursor level and protein group level. The Top3 method, which is the average MS signal response of the three most intense tryptic peptides of each identified protein, was used to plot the quantity of the targeted proteins in the samples [40, 41].

The generated mass spectrometry proteomics data have been deposited to the ProteomeXchange Consortium via the PRIDE partner repository with the dataset identifier PXD064428 [42]. DIA-NN is freely available for download from https://github.com/vdemichev/DiaNN.

Differences in protein expression between time points were evaluated in Python by comparing the log10 percent abundance. Protein distributions were compared using the Mann–Whitney U test in SciPy [43]. Expression levels of individual enzymes were compared using a t-test in SciPy and visualized using the Statannotations package [44].

## Results

### Evaluating the IPP-bypass pathway for expression and isoprenol production by *R. toruloides*

**Fig. 2:**
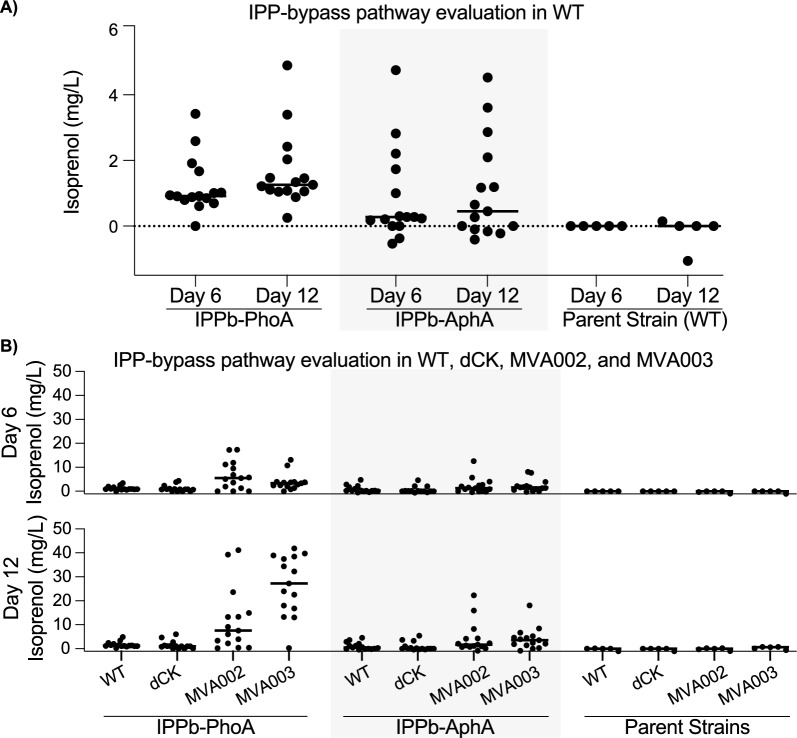
Isoprenol titers from the IPP-bypass pathways. **A** Individual titers from 15 distinct colonies of the IPP-bypass pathway transformed into wild-type *R. toruloides* and individual titers from 5 wild-type colonies. The line represents the median of each dataset. **B** Individual titers from 15 colonies of each transformation and 5 parent colonies of the IPP-bypass pathway transformed into wild type, dCK, MVA002, and MVA003. The lines in both parts represent the median of each dataset.

We first sought to determine if *R. toruloides* can produce isoprenol using the IPPb pathway previously demonstrated in *E. coli* and *S. cerevisiae*. Wild-type *R. toruloides* was transformed through *Agrobacterium*-mediated transformation (ATMT) with a plasmid containing a mevalonate kinase from *S. cerevisiae* (ERG12*sc*) off of a GAPDH promoter (MK; UniProt ID P07277), a mutant mevalonate pyrophosphate decarboxylase (PMD) off a TEF1 promoter [45], and either an Alkaline phosphatase from *E. coli* (PhoA, UniProt ID P00634) or a class B acid phosphatase from *E. coli* (AphA, UniProt ID P0AE22) off a GAPDH promoter. Due to the variability in integration location and copy number with this methodology [46, 47], 15 independent colonies from each transformation were evaluated for isoprenol production. Isoprenol titers were evaluated after 6 and 12 days of growth at 22 °C in a sugar-rich medium containing both glucose and xylose. Both pathways successfully produced isoprenol in *R. toruloides,* with a maximum of 4.9 mg/L after 12 days of cultivation (Fig. 2A).

We proceeded to explore metabolic engineering strategies to improve isoprenol production in *R. toruloides*. For *S. cerevisiae*, knocking out an endogenous choline kinase (CK) greatly increased isoprenol titers [11]. We identified an annotated enzyme (MycoCosm Protein ID: RTO4_15982) as having a 23.6% UniProt sequence similarity to CK. We used lithium acetate transformation to generate a RTO4_15982 knockout strain of *R. toruloides* (dCK) by replacing the coding sequence with a hygromycin resistance cassette. As an alternative approach, we sought to determine if increasing flux through the mevalonate pathway would improve production. A mevalonate overproduction strain (MVA001, Additional File 1 Fig. 1) was generated through the attempted introduction of ERG10, ERG13, and HMGR from *S. cerevisiae* using ATMT. MVA001 was qualitatively selected after ATMT based on an increased red appearance that likely indicated increased flux to mevalonate derived carotenoids. Metabolomic analysis confirmed that MVA001 produces 11.3 × as much mevalonate over wild-type *R. *toruloides (Additional File 1 Fig. 1A/B). MVA001 then underwent marker recycling to remove hygromycin resistance introduced during transformation to generate MVA002 allowing for these two strategies to be combined by using lithium acetate transformation to knock out RTO4_15982 in MVA002 to generate MVA003. Interestingly, proteomics analysis on MVA002 and MVA003 indicates while ERG13 and HMRG could be detected, ERG10 was not (Additional File 1 Fig. 1C). Full genome sequencing on MVA003 (Plasmidsaurus, Louisville, KY, USA) showed that only ERG13 and HMGR were successfully integrated into the genome (Additional File 3) highlighting the random nature of ATMT.

The two IPPb pathways containing either PhoA*ec* or AphA*ec* were transformed into dCK, MVA002, and MVA003 using ATMT, and the resulting colonies were screened as before at 6- and 12-day post-inoculation. Higher titers for each colony were generally observed after 12 days of cultivation (Additional File 1 Fig. 2). MVA002 and MVA003 increased isoprenol production as the 25th percentile, median, 75th percentile, and maximum titers were higher than in those produced in the WT background (Additional File 1 Fig. 2B). dCK, however, did not have a noticeable improvement in range or median over wild type (Fig. 2B/ Additional File 1 Fig. 2). By increasing flux to mevalonate, the highest producing strains in MVA002 and MVA003 reached maximum titers of 41.1 mg/L and 41.9 mg/L respectively.

### Evaluating pathway protein expression

A common issue for bioproduction is low expression of the desired pathway enzymes. This issue is particularly relevant for ATMT transformations as the number of integrated copies of the exogenous pathway is variable between colonies. Thus, we performed proteomic analysis to measure both endogenous and heterologous protein expression of transformed colonies to verify expression.

Samples of each IPPb PhoA expressing strain were collected after 6 and 12 days of cultivation. Proteomics was performed by mass spectroscopy and the Top3method was used to find the average MS signal response of the three most intense tryptic peptides to determine the quantity of the targeted proteins in the samples. The distribution in protein abundance was compared between the samples collected at day 6 and day 12. Endogenous protein expression was distinct between the two samples (p-value = 0.00, Additional File 1 Fig. 3A). However, when examining just the enzymes involved in the IPPb pathway there was no significant difference in the overall distribution (*p*-value = 0.825, Additional File 1 Fig. 3B). The heterologous proteins show no significant difference in expression between the two time points (*p*-value > 0.05 for all endogenous proteins, Additional File 1 Fig. 3C). We therefore proceeded to perform proteomics only on samples collected on day 6 to examine the expression of the introduced pathway enzymes.

**Fig. 3:**
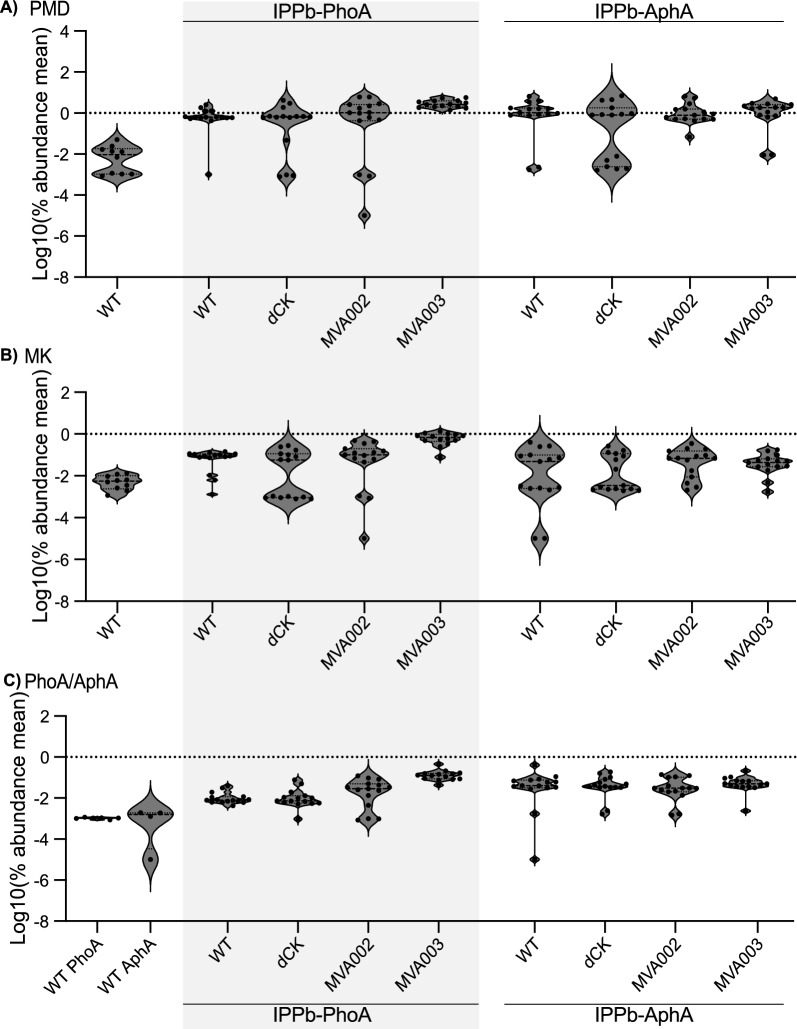
Expression of transformed pathways in 15 screened colonies from each transformation by proteomics performed on day 6 samples. **A** PMD expression, **B** ERG12 expression, **C** PhoAec or AphAec expression as indicated

At day 6, there was a large spread in protein expression across colonies. Of the heterologous proteins, PMD represented the largest portion of the proteome, in some instances reaching above 6% of the proteome (Fig. 3A). ERG12 (MK) and PhoA/AphA expression was much lower (Fig. 3B/C). The poor expression of PhoA, in particular, brought into question if the final phosphatases were functioning as expected (Fig. 3C).

### Exploring phosphatase modifications and alternatives

**Fig. 4:**
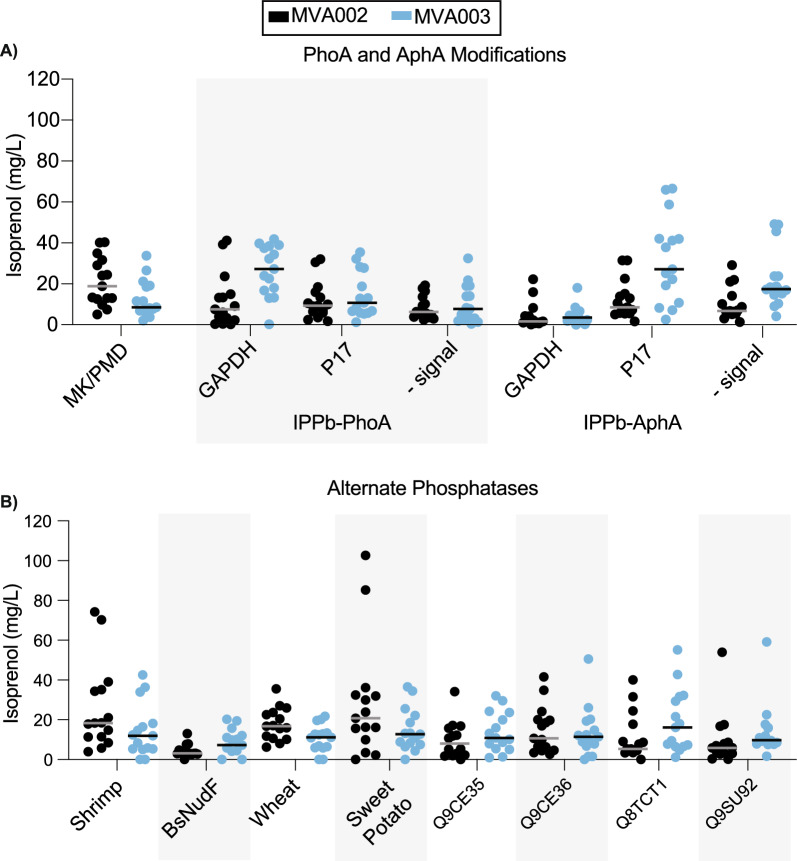
Isoprenol titers from the IPPb pathway with **A** alterations to the PhoA or AphA constructs or **B** alternative terminal phosphatases. Each point represents individual titers from 15 distinct colonies screened from each transformation. The horizontal lines represent the median of each dataset. Black points represent transformations in the MVA002 background, whereas blue represents transformations into the MVA003 background

To determine if PhoA*ec* or AphA*ec* were affecting isoprenol titers, MVA002 and MVA003 were transformed with MK and PMD without an additional phosphatase. The range in production of the MK/PMD transformed strains heavily overlapped with strains that contained PhoA*ec* or AphA*ec*, suggesting minimal isoprenol production activity by PhoA*ec* or by AphA*ec* (Fig. 4A).

In an attempt to increase PhoA*ec* and AphA*ec* expression and activity, the initial GAPDH promoter was replaced by one of the strongest observed promoters for *R. toruloides*, the P17 promoter [34]. Additionally, PhoA*ec* and AphA*ec* are predicted by SignalP V5 to have periplasmic signaling peptides at the N-termini [48] that could be diverting these enzymes away from isoprenol production. Plasmids containing versions of each enzyme without these signal sequences were generated to evaluate the effect of removing this signal sequence on isoprenol titers. These changes had little impact on the titers for the PhoA*ec* expressing pathways. Both modifications, however, increased the median and maximum measured titers for the AphA*ec* expressing pathways, particularly in the MVA003 background (Fig. 4A).

Given the random nature of the transformation method, it is challenging to conclude if the altered pathways directly caused the increased titers observed; however, the increased shift in the range of values provided hope that titers could be more dramatically improved by evaluating alternate phosphatases for isoprenol production. Eight alternative phosphatases were evaluated for isoprenol production in *R. toruloides*. The additional phosphatases included an ADP-ribose pyrophosphatase from *Bacillus subtilis* (denoted as BsNudF; UniProt ID P54570) that exhibited the highest isoprenol production in *E. coli* [49] and a purple acid phosphatase from *Triticum aestivum* (denoted as Wheat; UniProt ID C4PKK8) that had been successful in cell-free systems for isoprenol production [50]. Four other previously unevaluated enzymes from distant eukaryotic sources were also screened: a purple acid phosphatase from *Ipomoea batatas* (denoted a Sweet potato; UniProt ID Q9LL81), an alkaline phosphatase from *Pandalus borealis* (denoted as Shrimp; UniProt ID Q9BHT8), a thiamine phosphate phosphatase-like protein from *Arabidopsis thaliana* (denoted by its UniProt ID Q9SU92), and a phosphoethanolamine/phosphocholine phosphatase from *Homo sapiens* (denoted by its UniProt ID Q8TCT1). Finally, two kinases from *Lactococcus lactis* (denoted by their UniProt IDs Q9CE36 and Q9CE35) were also evaluated. BsNudF, wheat, sweet potato, and shrimp were driven off of the P17 promoter, while Q0SU92, Q9TCT1, Q9CE36, and Q0CE35 were driven off the GAPDH promoter. There was no major shift in the medians or ranges from any of the transformations with MK, PMD, and the array of phosphates. While sweet potato and shrimp both had two colonies with titers higher than observed with the PhoA*ec* and AphA*ec* variants (Fig. 4B), these increased titers are likely attributable to random integration and variable copy numbers not to improved pathway activity.

### Isoprenol tolerance and metabolism by *R. toruloides*

**Fig. 5:**
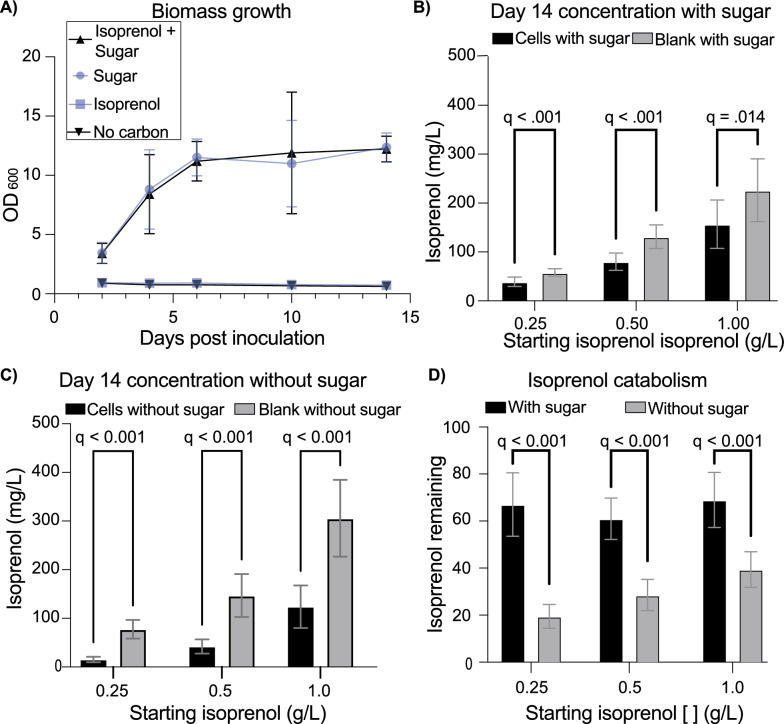
*R. toruloides* tolerance to and catabolism of isoprenol. **A** Growth of *R. toruloides* with and without sugar and/or isoprenol across 14 days of cultivation performed in triplicate. **B** Isoprenol concentration measured after 14 days of incubation in cultures containing glucose and xylose with and without *R. toruloides*. **C** Isoprenol concentration measured after 14 days of incubation in cultures without glucose and xylose with and without *R. toruloides*. **D** Isoprenol remaining when cells were present normalized to the no cell condition after 14 days of cultivation with and without sugar. For all graphs, points or bars represent the mean and error bars represent the standard deviation of three experimental replicates performed in triplicate. **B**-**D** were performed three times in triplicate. Significance (*q*-values) calculated in GraphPad Prism using a multiple unpaired t-test.

Along with low pathway flux, two additional factors may be contributing to the low titers we observe in *R. toruloides*: low tolerance to isoprenol and cell mediated degradation of isoprenol. A tolerable concentration of isoprenol for *R. toruloides* growth was determined by comparing the growth of wild-type *R. toruloides* in a sugar-rich medium containing initial concentrations of 0, 1, 5, or 10 g/L of isoprenol. *R. toruloides* grew successfully in the presence of 1 g/L isoprenol, over tenfold higher than the highest measured isoprenol titers (Supplementary Fig. 1; Fig. 5A).

Next, *R. toruloides* growth on isoprenol as a sole carbon source was evaluated using an equivalent medium containing 1 g/L isoprenol, but no glucose or xylose. No growth was observed using 1 g/L of isoprenol indicating that *R. toruloides* cannot use isoprenol as a sole carbon source to support growth (Fig. 5A). However, *R. toruloides* may still degrade isoprenol in a way that makes it challenging to produce.

To determine if *R. toruloides* degrades isoprenol, *R. toruloides* was grown in the sugar-rich medium containing three different concentrations of isoprenol: 0.25, 0.5, and 1 g/L. As isoprenol is volatile, matched controls without cells were used for comparison. When comparing these two conditions, there was up to a 28.2 ± 15.9% decrease in isoprenol concentrations in the sugar-rich medium supporting *R. toruloides* growth (Fig. 5B). To see if this effect was dependent on cell growth, the effect of *R.* toruloides on isoprenol concentrations was similarly evaluated without sugar to support growth. Under these conditions there was a greater reduction in isoprenol concentrations despite the lack of cell growth, up to an 80.1 ± 7.4% (Fig. 5C). At all three concentrations, there was a greater reduction in isoprenol when cells were present without sugar to support growth (Fig. 5D). This effect appears to be driven by *R. toruloides* as this effect was not observed when comparing the concentrations in the sugar-rich medium to the medium without sugar (Additional file 1 Fig. 4). Cumulatively, these results suggest that while *R. toruloides* can tolerate more isoprenol than it is producing currently, some form of degradation could be contributing to a decrease in overall titers.

### Isoprenol production by *R. toruloides* from sorghum-derived hydrolysates

We next wanted to explore isoprenol production in conditions more relevant to a biomanufacturing setting by testing the production of top producers in sorghum-derived hydrolysates. The highest isoprenol producing colony from across the strain backgrounds (WT, dCK, MVA002, and MVA003) of each pathway variant (e.g., IPPb-AphA*ec*) was reevaluated in a single experiment to directly compare isoprenol titers across the screens (Additional File 1 Fig. 5). The three strains with the highest mean production from this rescreen were chosen for testing in biomass derived cultivations: MVA003 with the IPPb pathway containing AphA*ec* with the signal sequence removed (MVA003_IPPb_AphA_-signal), MVA002 with only MK and PMD transformed (MVA002_IPPb_MK/PMD), and MVA002 with the IPPb pathway with the sweet potato purple acid phosphatase (MVA002_IPPb_SweetPotato).

**Fig. 6:**
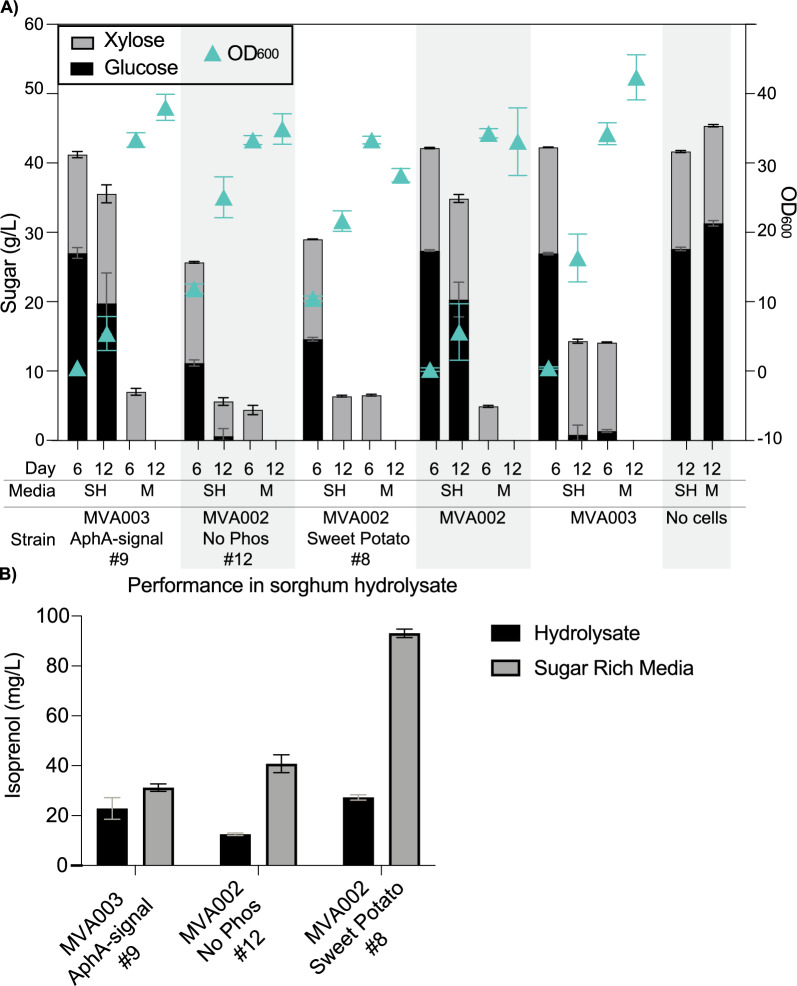
Isoprenol production in sorghum hydrolysates. **A** Concentrations of glucose (black) and xylose (gray) 6 and 12 days into cultivation. Green triangles represent the OD_600_ measured. **B** Isoprenol titers measured on day 12 in sorghum hydrolysate (black) and a corresponding sugar medium (gray). All experiments were performed in triplicate, the bars represent the mean, and the error bars represent the standard deviation

These top three performers were grown in sorghum-derived hydrolysate and a mock sugar-rich medium in parallel. The sorghum hydrolysates contained 41.5 ± 5.9 g/L of glucose and 17.0 ± 1.4 g/L of xylose, therefor the sugar-rich medium was modified to match these concentrations. Under these conditions in mock medium after 12 days of cultivation MVA003_IPPb_PhoA_-signal produced 31.2 ± 1.5 mg/L, MVA002_IPPb_MK/PMD produced 40.8 ± 3.6 mg/L, and MVA002_IPPb_SweetPotato produced 93.1 ± 1.7 mg/L. All three strains demonstrated slower growth, slower sugar consumption, and lower isoprenol production in hydrolysates compared to the mock medium (Fig. 6) with MVA003_IPPb_AphA_-signal producing 22.8 ± 4.3 mg/L, MVA002_IPPb_MK/PMD producing 12.5 ± 0.5 mg/L and MVA002_IPPb_Sweet_Potato producing 27.3 ± 1.1 mg/L in sorghum hydrolysates (Fig. 6B).

## Discussion

In this study, we successfully demonstrated isoprenol production by *R. toruloides* using the IPP-bypass pathway. While the initial titers established in this work are drastically lower than observed in more model organisms, the advantage of using this alternate host is demonstrated by successful production in sorghum-derived hydrolysates. Additionally, we identified a few key areas of engineering that could improve production in future work: (1) reduction of isoprenol degradation and (2) yeast specific enzyme optimization of the IPP-bypass pathway.

While increasing flux to the mevalonate pathway is a potential option for increasing isoprenol titers, our work suggests initially focusing further along in the pathway as there is still an apparent bottleneck within the IPP-bypass pathway and/or at the accumulation of isoprenol. The titers achieved here were drastically lower than had been expected given previous success with bioproduction through the endogenous mevalonate pathway in *R. toruloides*. Overexpression of bisabolene synthase alone in *R. toruloides* resulted in titers of up to 2.2 g/L of bisabolene (~ 22 × what we produce here of isoprenol), which requires two mevalonate precursors to generate compared to only one for isoprenol [25]. This discrepancy suggests to us that there is an underutilization of the available mevalonate generated through the endogenous mevalonate pathway in the isoprenol production strains. Focusing on better utilizing this endogenous pool first will hopefully allow for a greater benefit when later increasing mevalonate flux. For bisabolene, increasing flux through the MVA pathway and significant media optimization improved bisabolene titers up to 20.8 g/L after already appreciable titers had been measured [32].

The failure of the IPP-bypass pathway to yield the amounts of isoprenol observed in other organisms is likely due to a myriad of factors. The enzymes currently in the IPP-bypass for isoprenol production relay on their promiscuous activity. These enzymes are non-specific and could be generating other compounds toxic through phosphorylation or dephosphorylation of other essential metabolites that make isoprenol production challenging in this system. Even with significant optimization, *S. cerevisiae* has only yielded titers up to ~ 4 × higher of isoprenol (383.1 mg/L) than observed here despite being a more heavily studied system with a well annotated genome and proteome [11]. A more thorough understanding of the host organism biology could greatly help in synthetic biology efforts by understanding how these enzymes are acting outside of the desired activity.

Along with a better understanding of how the enzymes are acting in our host systems, a stronger understanding of alcohol catabolism and degradation could also improve titers. Even though degradation of externally applied isoprenol was reduced when sugar was present, the effect of this degradation could be larger if isoprenol is being degraded while being intracellularly produced. Work in other systems supports this approach as the titers achieved in *P. putida* increased fourfold when degradation was knocked out [9]. Further investigation into these issues, with a heavy focus on the known alcohol dehydrogenases, should yield some clarity into whether further pathway optimization and strain engineering could improve titers to make *R. toruloides* a viable organism for isoprenol production at scale.

While many hosts are screened for the production of a plethora of products, the basic biological understanding underlying production for the majority of these products is largely uninvestigated. More thoughtful biological studies of the production organisms would allow for a more thoughtful host–product pairing in the future.

## Conclusions

These efforts present an initial evaluation of isoprenol production by *R.* *toruloides*. Our top ATMT generated strain produced titers of 93.1 mg/L in sugar-rich medium and 27.3 mg/L in sorghum-derived hydrolysate. However, this work also outlines multiple different areas to be further evaluated for strain improvement. The isoprenol measured appears to largely, if not entirely, be produced by the promiscuous activity of an intermediate enzyme (PMD) as none of the phosphatases evaluated in this work greatly increased production while increasing the available mevalonate pool did help drive isoprenol production. We also observed that isoprenol degradation is present and amplified in the absence of sugar. Overall, our findings demonstrate the feasibility of *R. toruloides* as a host for isoprenol production, highlight some of the current limitations, and provide guidance for future efforts to improve production in *R. toruloides*.

## Supplementary Information

Supplementary Material 1. Supplementary figures 1–5

Supplementary Material 2. Sequences files of the plasmids used in this work

Supplementary Material 3. Sequencing reads to MVA003

## Data Availability

The proteomics data included in this manuscript will be available through the PRoteomics IDEntifications Database with the project accession PXD064428. All other data are available upon request.
