# Supplementary material for: Evaluating isoprenol production using the IPP-bypass pathway in the oleaginous yeast Rhodosporidium toruloides
Source: Biotechnol Biofuels Bioprod. 2026 Mar 3;19:31. doi: 10.1186/s13068-026-02750-w (PMC13063562; doi:10.1186/s13068-026-02750-w)
Supplement: Supplementary file 1 — Supplementary Material 1. Supplementary figures 1–5 [file 13068_2026_2750_MOESM1_ESM.docx]

Codon usage used by Genscript for optimization

TTT 5.9 (27567) TCT 9.9 (45737) TAT 3.1 (14449) TGT 2.3 (10579)

TTC 27.2 (126416) TCC 20.3 (93998) TAC 18.5 (85757) TGC 9.1 (42135)

TTA 0.9 (4328) TCA 7.0 (32367) TAA 0.2 (1119) TGA 0.9 (4140)

TTG 11.9 (55431) TCG 35.3 (163813) TAG 0.7 (3413) TGG 12.7 (58866)

CTT 11.2 (51846) CCT 17.8 (82506) CAT 5.2 (24063) CGT 7.1 (32829)

CTC 51.2 (237583) CCC 21.5 (99736) CAC 17.1 (79467) CGC 29.4 (136487)

CTA 2.7 (12558) CCA 8.5 (39314) CAA 9.2 (42646) CGA 11.0 (50923)

CTG 16.1 (74823) CCG 25.2 (116857) CAG 26.7 (123704) CGG 12.1 (56245)

ATT 5.9 (27205) ACT 8.2 (38240) AAT 3.9 (18185) AGT 5.2 (24251)

ATC 27.1 (125896) ACC 19.6 (90795) AAC 20.7 (95902) AGC 15.9 (73817)

ATA 1.2 (5797) ACA 6.3 (29214) AAA 5.4 (24900) AGA 2.7 (12659)

ATG 16.1 (74596) ACG 23.4 (108369) AAG 36.5 (169479) AGG 10.3 (47943)

GTT 8.7 (40330) GCT 18.3 (85046) GAT 10.6 (49015) GGT 10.2 (47357)

GTC 38.6 (178988) GCC 34.2 (158634) GAC 42.7 (197975) GGC 34.7 (160778)

GTA 2.9 (13229) GCA 15.5 (71991) GAA 14.5 (67164) GGA 14.3 (66228)

GTG 10.8 (50189) GCG 38.9 (180382) GAG 47.6 (220886) GGG 11.3 (52262)

| Accession number | Plasmid |
| --- | --- |
| [JPUB_026704](https://public-registry.jbei.org/entry/26704) | IPPb_Q9SU92_R-toruloides |
| [JPUB_026703](https://public-registry.jbei.org/entry/26703) | IPPb_Q8TCT1_R-toruloides |
| [JPUB_026702](https://public-registry.jbei.org/entry/26702) | IPPb_Q9CE36_R-toruloides |
| [JPUB_026700](https://public-registry.jbei.org/entry/26700) | IPPb_Q9CE35_R-toruloides |
| [JPUB_026693](https://public-registry.jbei.org/entry/26693) | IPPb_PhoA_no_signal_r-toruloides |
| [JPUB_026692](https://public-registry.jbei.org/entry/26692) | IPPb_AphaNoSignal_r-toruloides |
| [JPUB_026684](https://public-registry.jbei.org/entry/26684) | IPPb_P17_PhoA |
| [JPUB_026683](https://public-registry.jbei.org/entry/26683) | IPPb_P17_AphA |
| [JPUB_026681](https://public-registry.jbei.org/entry/26681) | IPPb_P17_BSnudF |
| [JPUB_026680](https://public-registry.jbei.org/entry/26680) | IPPb_P17_SweetPotato |
| [JPUB_026678](https://public-registry.jbei.org/entry/26678) | IPPb_P17_Shrimp |
| [JPUB_026677](https://public-registry.jbei.org/entry/26677) | IPPb_P17_Wheat |
| [JPUB_026676](https://public-registry.jbei.org/entry/26676) | MK_PMD_R-toruloides |
| [JPUB_020086](https://public-registry.jbei.org/entry/20086) | pPA218 MK PMD(mut), aphA GO |
| [JPUB_020080](https://public-registry.jbei.org/entry/20080) | pPA212 MK PMD(mut), phoA GO |

Table 1. JBEI accession numbers for the plasmids used throughout this work

C)

Supplementary Figure 1. Intracellular mevalonate pathway intermediates in WT and MVA001 in GX5AS medium on A) day 4 of cultivation and B) day 7. MVA, mevalonate; MVAP, mevalonate 5-phosphate; MVAPP, mevalonate 5-diphosphate; IPP, isopentenyl diphosphate. The cultivations were performed in biological triplicates, bars represent the mean and the error bars represent standard deviations.

Supplementary Figure 2. A) Comparison of isoprenol titers on day 6 and 12. Points represent individual colony titers, the line connects the samples at the two distinct time points. B) Box plot showing the minimum, 25^th^ percentile, median, 75^th^ percentile and maximum titers representing the 15 colonies screened in figure 2.

Supplementary Figure 3. A) Distribution of log10 percent abundance of all detected proteins across all samples from screened IPPb-PhoA. Includes transformants in all 4 genetic backgrounds. P-value was found by performing a Mann Whitney U test to compare the distribution at 144 vs 288 hours. B) Distribution of log10 percent abundance of only pathway proteins. P-value was found by performing a Mann Whitney U test to compare the distribution at 144 vs 288 hours. C) Comparison of exogenous protein expression between time points. The p-value for each protein was calculated by performing a t-test comparing the log10 percent abundance at 144 (blue) vs 288 hours (orange).

Supplementary Figure 4. A) Growth of R. toruloides in a sugar rich medium with varying concentrations of isoprenol. Points represent the mean. The standard deviation is encompassed within each point. B) The mean and standard deviation of the isoprenol concentration measured after 14 days of incubation in cultures with and without sugar without cells. Both experiments were performed three times in triplicate. Significance (q-values) calculated in GraphPad Prism using a multiple unpaired t-test.

Supplementary Figure 5. Re-screening of all previous top hits in a mock medium to identify best performers across screens. Experiment was performed in triplicate with the bars representing the mean and the error bars representing the standard deviation.
